# Oral Elesclomol Treatment Alleviates Copper Deficiency in Animal Models

**DOI:** 10.3389/fcell.2022.856300

**Published:** 2022-04-01

**Authors:** Sai Yuan, Tamara Korolnek, Byung-Eun Kim

**Affiliations:** ^1^ Department of Animal and Avian Sciences, University of Maryland, College Park, MD, United States; ^2^ Biological Sciences Graduate Program, College Park, MD, United States

**Keywords:** copper transport, copper defficiency, intestine, Menkes disease, elesclomol, ATP7A copper exporter, CTR1 copper importer, *C. elegans*

## Abstract

Copper (Cu) is an essential trace element for key biochemical reactions. Dietary or genetic copper deficiencies are associated with anemia, cardiomyopathy, and neurodegeneration. The essential requirement for copper in humans is illustrated by Menkes disease, a fatal neurodegenerative disorder of early childhood caused by mutations in the ATP7A copper transporter. Recent groundbreaking studies have demonstrated that a copper delivery small molecule compound, elesclomol (ES), is able to substantially ameliorate pathology and lethality in a mouse model of Menkes disease when injected as an ES-Cu^2+^ complex. It is well appreciated that drugs administered through oral means are more convenient with better efficacy than injection methods. Here we show, using genetic models of copper-deficient *C. elegans* and mice, that dietary ES supplementation fully rescues copper deficiency phenotypes. Worms lacking either the homolog of the CTR1 copper importer or the ATP7 copper exporter showed normal development when fed ES. Oral gavage with ES rescued intestine-specific *Ctr1* knockout mice from early postnatal lethality without additional copper supplementation. Our findings reveal that ES facilitates copper delivery from dietary sources independent of the intestinal copper transporter CTR1 and provide insight into oral administration of ES as an optimal therapeutic for Menkes disease and possibly other disorders of copper insufficiency.

## Introduction

Copper (Cu) is an essential micronutrient required as a catalytic cofactor for a wide range of biochemical processes, including ATP generation in mitochondria, maturation of hormones, iron mobilization, and disproportionation of toxic superoxide (Kim et al., 2008). The essential nature of copper in humans is underscored by Menkes disease, an X-linked copper deficiency disorder caused by mutations in the ATP7A copper exporter (Kaler, 2011). Defects in Menkes patients are attributed primarily to impaired copper transport from intestinal epithelial cells (IECs) to the circulation, resulting in a systemic copper deficiency (Petris et al., 1996; Wang et al., 2012a). Clinical symptoms of Menkes include severe neurodegeneration, connective tissue defects, hypothermia, and impaired pigmentation. Most cases of Menkes disease typically lead to death within a few years after birth. Some individual patients harboring mild forms of ATP7A mutations exhibit extended life spans; nevertheless, profound neurodevelopmental delays are observed in these patients (Madsen and Gitlin, 2007; Kaler, 2011).

Yeast, fruit flies, zebrafish, and mice have all been used as model organisms to understand the role of ATP7A in copper metabolism and evaluate potential treatments for Menkes disease (Nose et al., 2006; Turski and Thiele, 2007; Madsen and Gitlin, 2008; Smith et al., 2017). Our recent studies in *C. elegans* models revealed that tight control of copper homeostasis is required for normal growth and development of worms, and that this regulation is in part controlled by the functions of a CTR1 copper importer ortholog, CHCA-1, and an ATP7A ortholog, CUA-1, in the intestine (Chun et al., 2017a; Yuan et al., 2018). Given the conservation of the pathway for the import and distribution of dietary copper in *C. elegans*, this animal is well-poised to serve as a model organism for therapeutic approaches for Menkes disease treatments.

The copper-binding molecule elesclomol (ES) was identified in a yeast-based screen as an efficient copper delivery agent for cellular copper-dependent proteins. ES is especially efficient at delivering copper to mitochondrial cuproenzymes such as cytochrome c oxidase (CcO), which is essential for energy generation (Soma et al., 2018). We have recently shown that subcutaneous injections of an ES-Cu^2+^ complex in the *mottled-brindled* (*mo-br*) mouse, a model for classical Menkes disease (Rossi et al., 2001), resulted in profound improvement of Menkes-like pathology including neonatal lethality and neurodegeneration (Guthrie et al., 2020). The ES-Cu^2+^ complex injections rescued systemic copper levels in multiple tissues including the brain, which is highly susceptible to copper-deficiency, and restored activities of essential cuproenzymes to the point where catastrophic outcomes were prevented. However, it is not known whether orally supplied ES can escort copper through polarized enterocytes to be effective in treating the severe systemic copper deficiency found in animal models of Menkes disease or under conditions of complete *Atp7a* ablation.

In this study, we use genetic models of copper-deficient *C. elegans* to demonstrate that supplementation with ES alone in the diet fully rescues developmental defects found in *chca-1-* and *cua-1-*deleted worms. We further demonstrate that oral gavage with ES alone can rescue intestine specific *Ctr1* knockout mice from neonatal mortality without additional copper supplementation. These results suggest that ES can facilitate copper delivery from dietary sources independent of the intestinal copper transporting system and offers efficient administration options in treating Menkes and other copper-deficiency patients.

## Results

### ES Rescues Developmental Phenotypes and Copper Levels in *Chca-1-*Mutants in *C. elegans*

**FIGURE 1:**
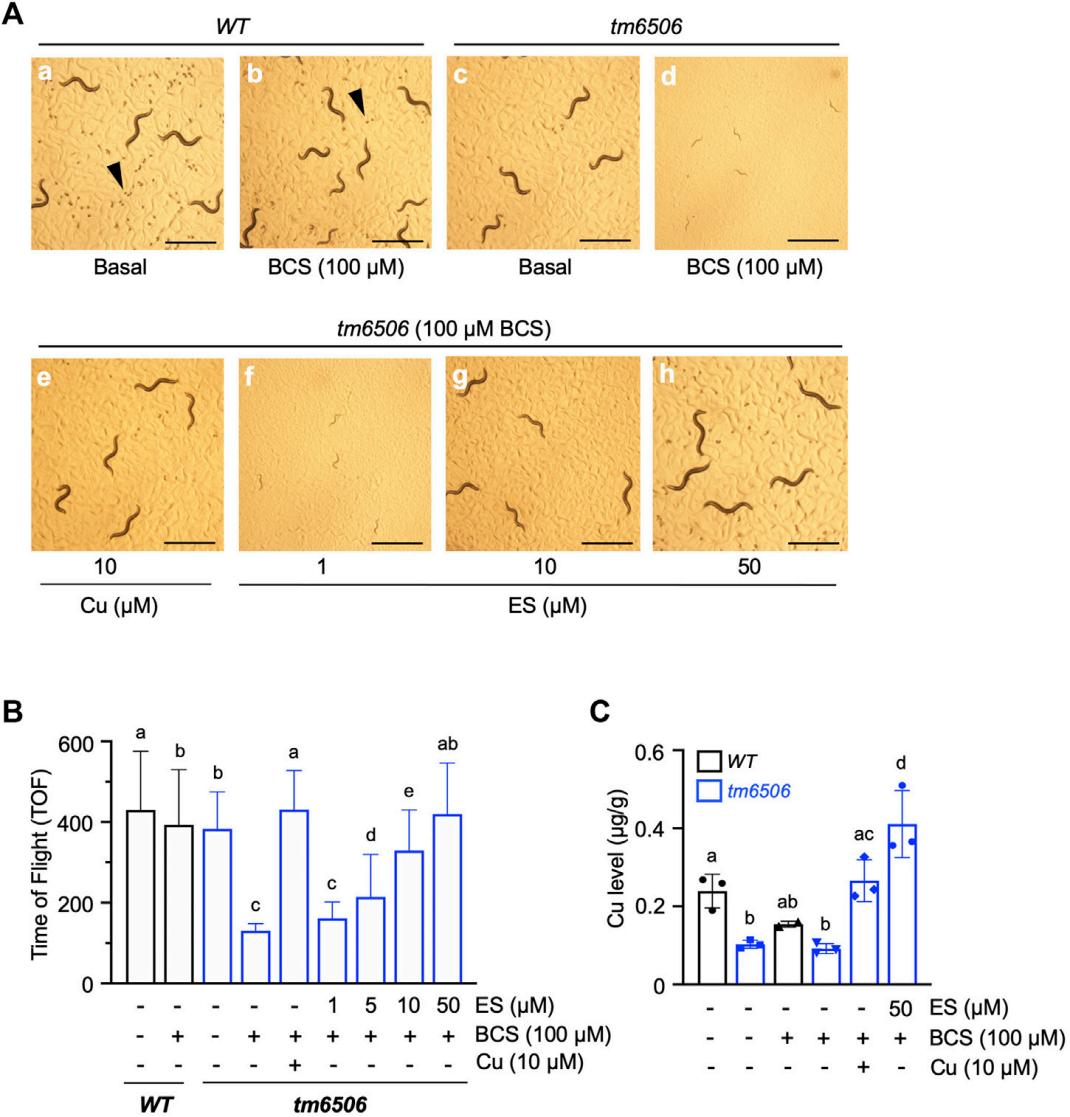
Dietary ES rescues larval arrest phenotypes associated with copper deficiency in *chca-1* mutants in *C. elegans.* **(A)**, Representative images of *C. elegans* nematodes grown under copper-deficient conditions. Synchronized L1 larval cultures of WT and *chca-1(tm6506)* mutant worms were seeded onto control plates, plates containing 100 *µ*M of the copper chelator BCS or BCS-containing plates supplemented with CuCl_2_ (10 *µ*M) or ES (from 1 to 50 *µ*M). Images taken 72 h after seeding worms. Arrowheads indicate eggs. Egg numbers in the representative fields are a: ∼110, b: ∼20, c:∼10, d: none, e: ∼10, f: none, g: none, h:∼50. Scale bar, 1 mm. **(B)**, Body length quantification as a measure of larval development. Worms were grown as in A. and body length was determined using a COPAS BioSort system. Number of worms tested for each condition: 88-269. Groups with different letters are statistically significantly different from each other at *p* = 0.05 (one-way ANOVA, Sidak post hoc test). **(C)**, Total worm copper levels measured by ICP-MS. Different letters indicate statistically significant differences as determined by a two-way ANOVA, Tukey post hoc test. Error bars represent mean ± SD.

We recently demonstrated that nematodes expressing a non-functional copy of intestinal CHCA-1, the nematode homolog of the mammalian copper transporter *Ctr1,* fail to accumulate wild-type levels of copper in their body and arrest during larval development when grown under copper deficient conditions (Yuan et al., 2018). Given the functional conservation between nematode CHCA-1 and mammalian *Ctr1,* its facile manipulation and genetic tractability, we set out to determine if *C. elegans* is a suitable animal model to explore ES as a potential therapeutic for copper deficiency disorders. To that end, we first asked if ES can rescue the copper deficiency phenotypes of the *chca-1(tm6506)* mutant strain that we previously characterized (Yuan et al., 2018). Synchronized L1 cultures of *chca-1(tm6506)*) mutants and wild-type (WT) worms were grown on standard growth media in the absence (Basal) or presence of 100 µM bathocuproinedisulfonic acid (BCS)—a membrane-impermeable copper chelator—or BCS-containing media supplemented with 10 µM copper chloride (CuCl_2_) or various concentrations of ES (from 1 to 50 µM). As shown in Figure 1A (panel d), *chca-1* mutants grown on BCS-containing plates have a dramatic larval arrest phenotype when compared to mutants grown on control plates (Figures 1A,C) and WT worms grown under either condition. As expected, this larval arrest phenotype is fully rescued by copper supplementation, and importantly, by supplementation with ES in a dose dependent matter (Figure 1A, e-h).

We used COPAS sorting to quantitatively examine the larval phenotype of the *chca-1(tm6506)* mutant. As shown in Figure 1B, rescue of the larval arrest phenotype—as determined by an increase in body size—is observed at doses as low as 5 µM ES with full rescue observed at 50 µM ES supplementation. Moreover, the ability of ES to rescue the larval phenotype is comparable to rescue observed by copper supplementation. Furthermore, ICP-MS analysis of worms grown under the same conditions demonstrated that whole animal copper levels in the *chca-1(tm6506)* are fully rescued by ES supplementation (Figure 1C). These results strongly suggest that ES can efficiently restore copper levels and reverse phenotypes caused by copper deficiency even in the presence of the high-affinity copper chelator, BCS. Importantly, these data suggest that *C. elegans* represents a viable experimental animal model to interrogate the biology of ES and its potential as a therapeutic for copper deficiency disorders.

### ES Supplementation Rescues the Developmental Phenotypes of a *C. elegans* Model of Menkes Disease

**FIGURE 2:**
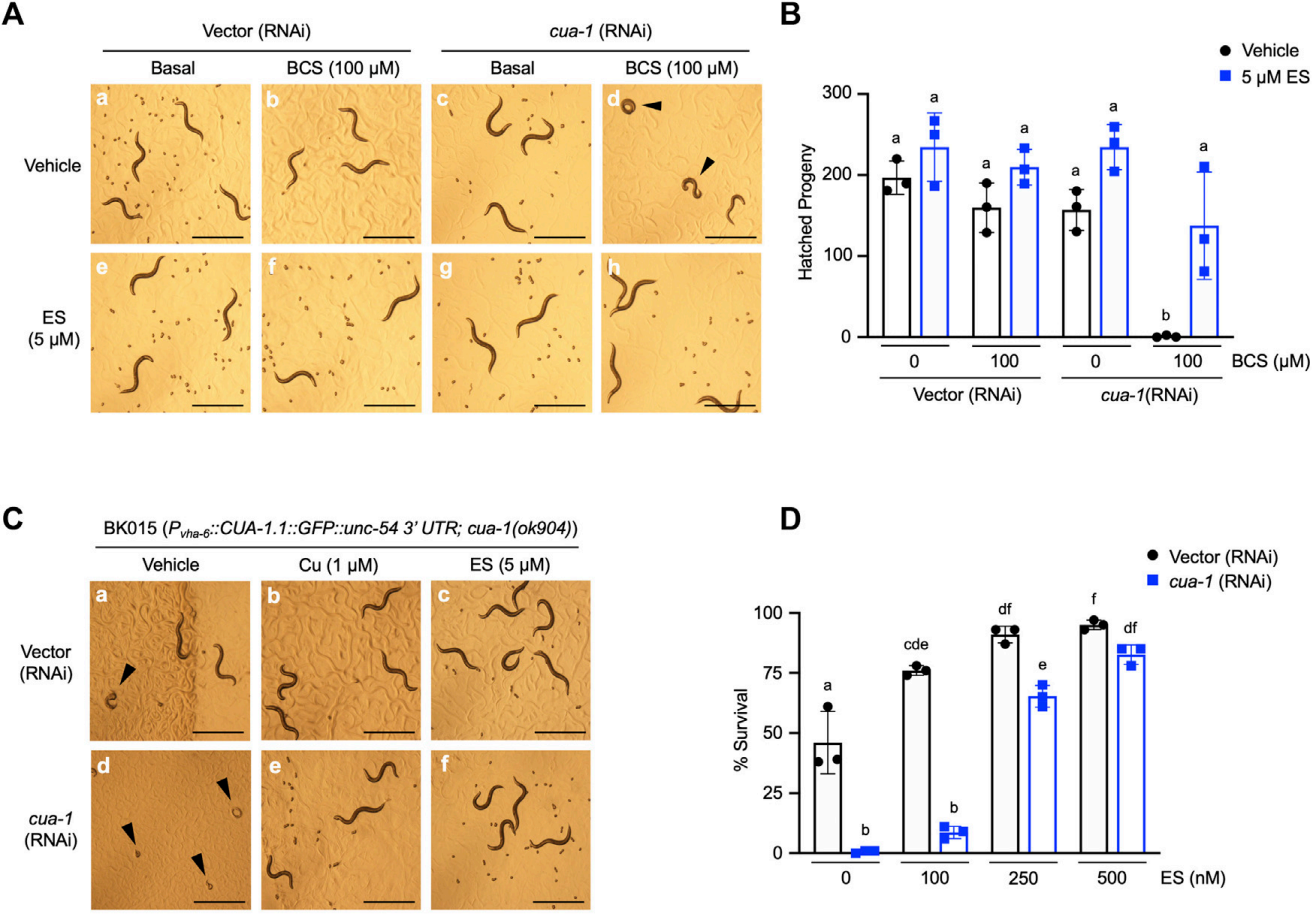
Dietary ES supplementation rescues developmental phenotypes in a *C. elegans* model of Menkes disease. **(A)**, (top row) WT worms were exposed to RNAi from the L1 stage for 4 days with or without 100 µM BCS treatment in the presence of vehicle (DMSO); (bottom row) worms grown under identical conditions as above supplemented with 5 µM ES. Representative microscope images from three independent replicates are shown; arrowheads show representative arrested worms. Scale bar, 1 mm. **(B)**, WT worms grown under varying RNAi and BCS conditions supplemented with vehicle (DMSO) or 5 µM ES were picked to individual plates at the L4 stage in triplicate, allowed to lay eggs, and transferred to fresh plates every 24 h for 3 days. Eggs were incubated overnight to allow hatching and progeny number was determined as the total hatched larvae, *n* = 3 biological replicates (different letters indicate statistical significance at *p* = 0.05, by two-way ANOVA, Tukey’s post hoc test). Error bars represent mean ± SEM. **(C)**, Transgenic worms expressing CUA-1 in the intestine [BK015 strain, *P*_*vha-6*_ *::CUA-1.1::GFP::unc-54 3*′*UTR; cua-1*(*ok904*)] were cultured on RNAi plates supplemented with either vehicle (DMSO), 1 µM copper, or 5 µM ES from the L1 stage for 4 days. Representative microscope images from three independent replicates are shown; arrowheads point at arrested larvae that eventually die. Scale bar, 1 mm. **(D)**, BK015 strain worms were cultured on RNAi plates supplemented with either vehicle (DMSO) or indicated amounts of ES from the L1 stage for 4 days and then scored for their ability to survive larval lethality and make it to the adult stage (% survival on *y*-axis). *n* = 3 biological replicates (different letters indicate statistical significance by two-way ANOVA, Tukey’s post hoc test). Error bars represent mean ± SEM.

Our previous studies identified *cua-1*, the *C. elegans* homolog of ATP7A/B, as a key regulator of systemic copper homeostasis in the worm (Chun et al., 2017a). While CUA-1 is expressed in the intestine, hypodermis, pharynx, and neurons in *C. elegans, cua-1* primarily functions as a copper exporter at the basolateral membrane of the intestine, delivering dietary copper to extraintestinal tissues as needed. Notably, *cua-1* mutant model Menkes disease in that they exhibit systemic copper deficiency (Chun et al., 2017a). *cua-1* RNAi in the presence of 100 µM BCS results in a severe developmental arrest phenotype (Figures 2A,D). Importantly, this phenotype is rescued by treatment with 5 µM ES even in the absence of exogenous copper supplementation (Figures 2A,H). Worms simultaneously grown in *cua-1(RNAi)* and in the presence 100 µM BCS that manage to escape arrest and reach reproductive age were interrogated for their ability to produce viable embryos as measured by the hatching rate. As shown in Figure 2B, *cua-1* RNAi in the presence of 100 µM BCS results in a severe defect in hatching rate. However, this phenotype can be partially rescued by treatment with 5 µM ES.

A number of groups have shown that *cua-1* is expressed in multiple tissues in the worm (Wakabayashi et al., 1998; Chun et al., 2017a). To specifically demonstrate ES rescue of copper transport across the intestinal membrane, we made use of the BK015 worm line [*P*_*vha-6*_ *::CUA-1.1::GFP::unc-54 3′UTR; cua-1*(*ok904*)] which ectopically expresses a GFP-tagged version of CUA-1 driven by an intestine-specific promoter in a *cua-1*-null mutant background (Chun et al., 2017a). BK015 worms require added dietary copper for optimal growth but can be maintained with about 50% survival on standard OP50 bacteria without dietary copper amendments (Figure 2C, a-b and D). This morbidity is presumably caused by the lack of CUA-1 in extraintestinal tissues such as pharynx and neurons. Knockdown of *cua-1* in BK015 worms results in complete larval lethality (Figures 2C,D). However, this lethality can be rescued almost fully in a dose-dependent manner by 500 nM ES supplementation (Figure 2D). Thus, ES is specifically capable of rescuing defective intestinal copper export, the major hallmark of Menkes disease, in a whole animal model. Notably, 5 µM ES also rescues suboptimal growth of BK015 under basal media conditions (Figures 2C,F), indicating that ES is capable of restoring copper homeostasis defects further downstream of intestinal copper export.

### Effects of ES on CUA-1 Localization in the Intestine

**FIGURE 3:**
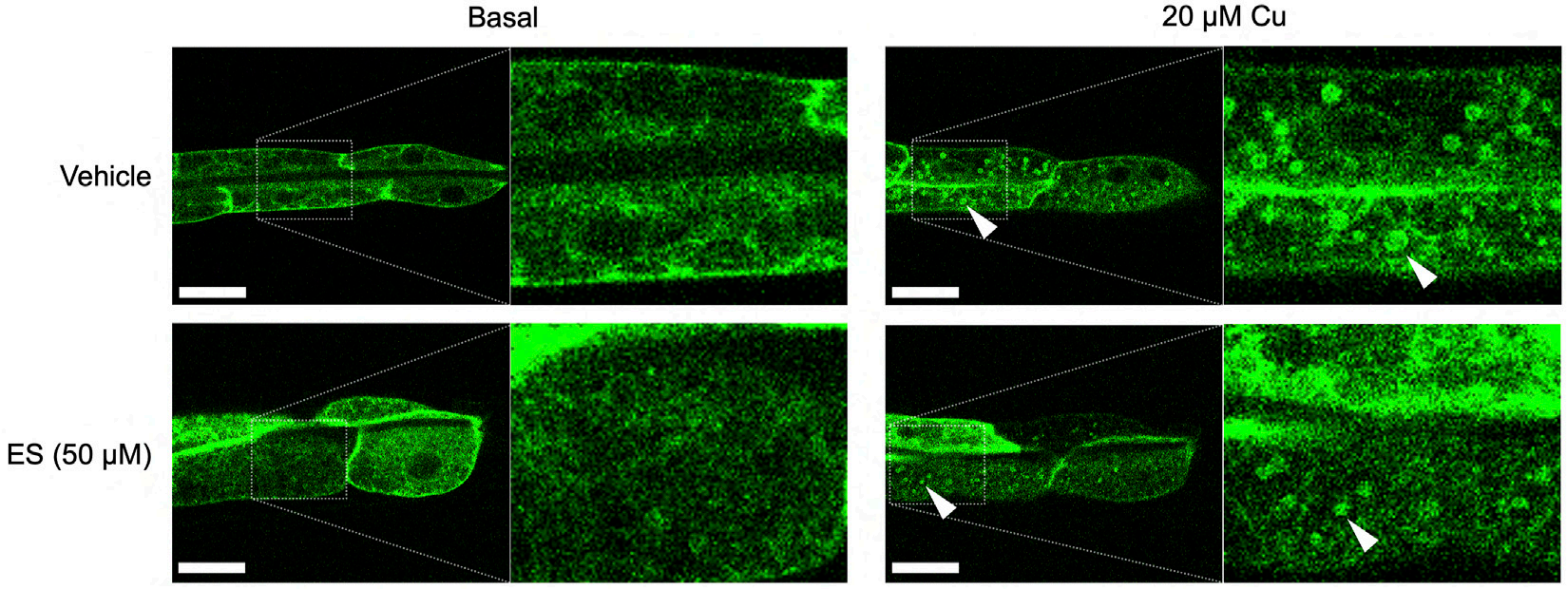
Treatment with ES does not affect subcellular localization of CUA-1 in the intestine. Synchronized BK014 transgenic worms (*P*_*vha-6*_ *::CUA-1.1::GFP::unc-54 3*′ *UTR*) were cultured on NGM agar plates with indicated amounts of CuCl_2_, vehicle (DMSO) or ES from the L1 stage. *CUA-1.1::GFP* localization in live L4 worms was examined using confocal microscopy. Representative microscope images from three independent replicates are shown; arrowheads indicate CUA-1 localized to gut granules upon copper treatment. Boxed images are enlarged and added as indicated with dotted lines. Scale bar, 20 µm.

To further assess the effect of ES on organismal copper homeostasis in *C. elegans*, we analyzed the subcellular localization of CUA-1.1, which is encoded by one of two splice isoforms of *cua-1*. We have previously shown that CUA-1.1 localizes to the basolateral membrane of the worm intestine under basal conditions. In response to elevated amounts of dietary copper, CUA-1.1 redistributes to lysosome-like gut granules in intestinal cells, likely as a protective measure to sequester potentially toxic excess copper. The other splice variant, CUA-1.2, localizes constitutively to the basolateral membrane irrespective of dietary copper concentration (Chun et al., 2017a). Localization of CUA-1.1 can be perturbed genetically, as loss of *chca-1* decreased distribution of CUA-1.1 to granules, indicative of copper deficiency even in the presence of sufficient dietary copper (Yuan et al., 2018). Surprisingly, ES treatment did not appear to influence the subcellular distribution of CUA-1.1 in the gut (Figure 3). This result is intriguing and may suggest an alternative copper uptake pathway exploited by ES that potentially preserves copper in a less toxic form that does not trigger CUA-1.1 re-localization to gut stress granules.

### Oral Administration of ES Rescues Neonatal Lethality and Growth Defects in Intestine-specific *Ctr1* Knockout Mice

**FIGURE 4:**
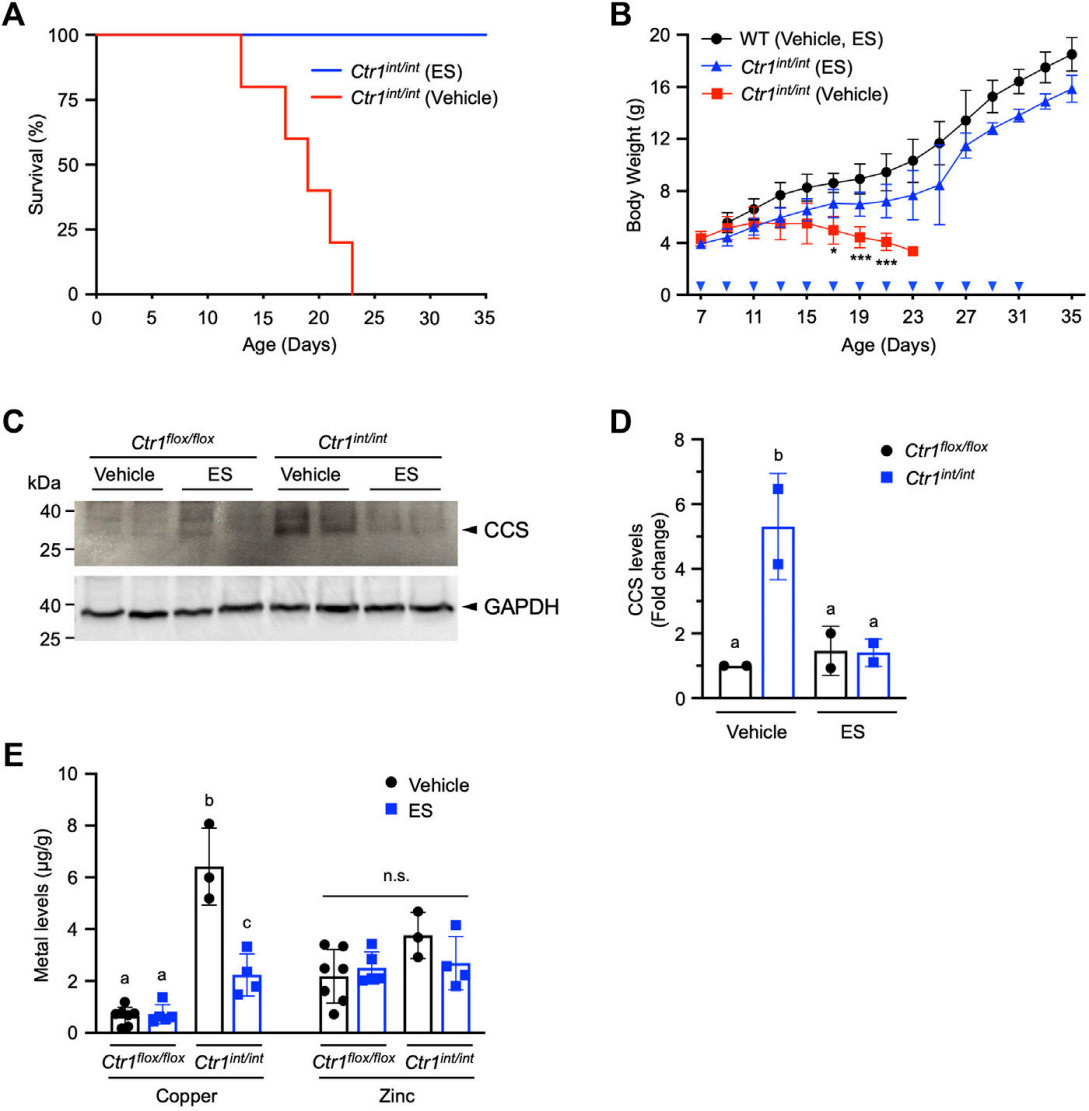
Rescue of *Ctr1* ^*int/int*^ mice with ES by oral gavage. *Ctr1* ^*int/int*^ and control littermates were given ES or vehicle treatment by gavage every 2 days until PND 31. **(A)**, Kaplan-Meier plot showing percent survival of *Ctr1* ^*int/int*^ administered vehicle (red, *n* = 5), or ES (blue, *n* = 4). **(B)**, Body weight (g) of control littermates (*Ctr1* ^*flox/flox*^ or *Ctr1* ^*flox/+*^ referred to as WT), or *Ctr1* ^*int/int*^ treated with vehicle, or ES. Cohorts consisted of WT (Vehicle or ES, *n* = 4), *Ctr1* ^*int/int*^ (Vehicle, n = 5), and *Ctr1* ^*int/int*^ (ES, *n* = 7). No significant difference in growth between WT vehicle and WT ES was found (data not shown) as tested in an ES injection experiment (Guthrie et al., 2020). Asterisk marks indicate significant difference compared to the *Ctr1* ^*int/int*^ vehicle group (two-way ANOVA, Tukey’s post hoc test, *p < 0.05, ***p < 0.001). Error bars represent mean ± SD. **(C)**, SDS-PAGE analysis of total extracts of intestinal epithelial cells (IECs) from two representative *Ctr1* ^*flox/flox*^ and *Ctr1* ^*int/int*^ mice (PND 17) 10 days after commencing treatment. CCS levels in IECs were detected by immunoblotting. CCS and GAPDH (used as a loading control) bands are indicated with arrowheads. **(D)**, Quantification of relative CCS expression (normalized by GAPDH) in the enterocytes of the *Ctr1* ^*flox/flox*^ and *Ctr1* ^*int/int*^ mice. Groups with different letters are statistically significantly different from each other at *p* < 0.05 (one-way ANOVA, Sidak post hoc test). Error bars indicate mean ± SD. **(E)**, Copper and zinc levels in *Ctr1* ^*int/int*^ IECs after ES or vehicle administration (Figure 4C). Groups with different letters are significantly different from each other at *p* = 0.05. n.s., not significant (one-way ANOVA, Sidak post hoc test). Error bars represent mean ± SD.

We next aimed to determine if ES was capable of escorting copper across the polarized intestinal epithelium in mammals. Loss of intestinal *Ctr1* in mice leads to profound neonatal defects in copper delivery to peripheral tissues, and results in drastically stunted development with mutants exhibiting 100% lethality within 25 days after birth (Nose et al., 2006). Intestine-specific *Ctr1* knockout mice (*Ctr1* ^*int/int*^) were administered a 10 mg/kg dose of ES by oral gavage once every 2 days from postnatal day (PND) 7. We found a 35-days survival rate of 100% in ES-treated *Ctr1* ^*int/int*^ mice, whereas all mutant mice treated with vehicle died between PND 12 and 23 (Figure 4A). ES-treated knockout pups showed normal development and weight gain with no apparent difference as compared to vehicle- or ES-treated WT mice, while all vehicle-treated *Ctr1* ^*int/int*^ pups failed to thrive, lost weight, and began dying around PND 12 (Figure 4B). These results suggest that ES can deliver available copper from normal dietary sources—namely, milk from mothers fed a regular chow diet.

To further characterize the intestinal phenotype of ES-rescued *Ctr1* ^*int/int*^, we isolated IECs from PND 17 mice treated with ES or vehicle, and evaluated the abundance of copper chaperone for superoxide dismutase (CCS), a marker for intracellular copper-availability, as it is known that CCS levels are inversely proportional to cytosolic copper bioavailability (Bertinato and L'Abbé, 2003; Kim et al., 2010). As expected, we observed increased CCS steady-state levels in vehicle-treated *Ctr1* ^*int/int*^ intestinal cells as compared to levels in cells from control littermates (*Ctr1* ^*flox/flox*^) treated with vehicle or ES. The increased CCS levels in cells from ES-treated *Ctr1* ^*int/int*^ were suppressed back to levels observed in *Ctr1* ^*flox/flox*^ mice (Figures 4C,D), indicating that the limited copper availability in the *Ctr1*-deleted enterocytes was normalized by ES gavage. Hyperaccumulation of copper in the IECs observed in *Ctr1* ^*int/int*^ (Nose et al., 2006) was also rescued in the ES-treated mutants, while ES did not influence overall zinc levels in the intestine (Figure 4D) suggesting a copper-specific function for ES. Taken together, our results indicate that oral treatment with ES is capable of escorting copper across a CTR1-deficient polarized enterocyte layer.

## Discussion

*C. elegans* has emerged as a highly amenable model of micronutrient metabolism for metals such as iron, heme-iron, copper, and zinc (Anderson and Leibold, 2014; Sinclair and Hamza, 2015; Dietrich et al., 2017). Our recent studies revealed that CUA-1 is a key intestinal copper exporter in the worm that plays an analogous role to the human P-type ATPase in controlling systemic copper homeostasis to the human P-type ATPases (Chun et al., 2017a; Pierson et al., 2018). Moreover, we found that worms lacking CHCA-1, a CTR1 ortholog, exhibit impaired copper acquisition and profound growth and reproductive defects under copper-limiting conditions, suggesting that the pathway for intestinal copper uptake *via* the conventional CTR1-ATP7A axis is conserved in worms (Wakabayashi et al., 1998; Yuan et al., 2018).

In this study, we show that low concentrations of ES without additional copper supplemented in the media can almost fully rescue the phenotypes caused by either the loss of CHCA-1 or CUA-1 (Figure 1 and Figure 2). We observed that ES is capable of increasing copper accumulation even in the presence of BCS. Considering that ES-Cu^2+^ is much more stable than BCS-Cu^2+^ (Xiao et al., 2008), it is possible that ES binds Cu^2+^ from dietary sources, forms a highly stable and membrane permeable complex, and mediates copper delivery from the intestinal lumen in the form of ES-Cu^2+^. It is unknown how copper is liberated from the ES-Cu^2+^ complex to be integrated into various cuproproteins, though we do not rule out the possibility that ES is somehow metabolized intracellularly and then copper is released. Future mechanistic studies will be required to answer these questions.

While it is unknown how copper is transported to and utilized by different subcellular compartments after ES facilitates its entry into intestinal cells, our results in the BK015 strain treated with *cua-1* RNAi show that ES treatment can fully rescue the lethality in worms lacking *cua-1* expression across all tissues in the worm, suggesting that *cua-1*-independent copper delivery from the intestine to other tissues can be mediated by ES. These results indicate that ES can bypass the genetic blockage of intestinal copper absorption and supply copper to circulation and peripheral tissues in *C. elegans*. Additionally, the findings of this study suggest that *C. elegans* can be exploited as an animal model for screening of pharmacological chaperones for cellular copper distribution.

ES-Cu^2+^ administration by subcutaneous injections resulted in substantial rescue of lethal phenotypes in the *mo-br* mouse by restoring critical copper-dependent enzymes such as cytochrome c oxidase in the mitochondria (Guthrie et al., 2020). However, the efficiency of ES-mediated copper delivery to cuproenzymes, particularly in the secretory pathway, seemed to be limited. For example, coat pigmentation, which is dependent on tyrosinase in the secretory compartment that requires ATP7A for its metalation in the Golgi (Petris et al., 2000), was only marginally rescued by ES-Cu^2+^ administration in these experiments (Guthrie et al., 2020). A number of copper dependent enzymes found in the secretory pathway are conserved in *C. elegans*, including tyrosinase (Sendoel et al., 2010), and neuropeptide amidation enzymes (Van Bael et al., 2018; Yuan et al., 2018). Establishing reporter lines for these enzymes would be a first step in exploring and testing subcellular targeting efficiency of structural analogues of ES or new forms of chemical copper ionophores and chaperones in a worm model, ultimately leading to more effective pharmacological treatment of copper deficiency in all tissues and subcellular compartments.

In this study, oral treatment of *Ctr1* ^*int/int*^ mice with ES rescued the lethality of these mutants and, notably, this rescue occurred in the absence of additional dietary copper. Similar to the *mo-br* mice, *Ctr1* ^*int/int*^ mice exhibit copper deficiency in peripheral tissues due to impaired intestinal copper absorption. These results are directly relevant to long-term therapeutic applications expected in treating disease of copper deficiency including Menkes patients, as oral treatment with ES would minimize invasive procedures. Menkes disease typically presents in the first few months after birth and results in death by 3 years of age. This indicates a high demand for copper during the early developmental stages of mammals (Prohaska and Brokate, 2002) and underscores the critical importance of early and long-term treatments for Menkes patients (Kaler, 2011). Our results in the *cua-1*-depleted worm model suggest the possibility of ATP7A-independent intestinal copper export by ES, and it is worthwhile to further test the efficacy of ES oral administration in mammalian Menkes models. An important follow up question would be whether ES (or an ES-Cu^2+^ complex) could function as an efficient copper delivery agent to the fetus via placental membranes. This question would be readily testable in future studies with genetic mouse models of copper deficiency, including conditional *Atp7a* knockouts (Wang et al., 2012a; Wang et al., 2012b), by feeding ES to pregnant mice.

An interesting observation in ES-treated *Ctr1* ^*int/int*^ mice is that ES rescued the hyperaccumulation of copper in the intestine (Figure 4). While it is unknown exactly how *Ctr1*-deleted IECs accumulate elevated levels of copper (Nose et al., 2006), it is possible that copper overaccumulation occurs in vesicular compartments, where CTR1 normally functions to mobilize copper from endosomes to the cytosol in a CTR2-dependent process. Hyperaccumulation of copper also occurs in *Ctr2* knockout fibroblasts and tissues in CTR2 knockout mice (Öhrvik et al., 2013; Öhrvik and Thiele, 2015; Öhrvik et al., 2016). Further studies will be necessary to establish whether ES can be used as a valuable tool for investigating subcellular copper homeostasis, mediated potentially by vesicular copper sequestration (Leary and Ralle, 2020). Taken together, our results from the rescued *chca-1* mutant worm, *Ctr1* ^*int/int*^ mice, and the Menkes worm model provide novel insights into using the copper binding molecule ES or its potential analogs to treat Menkes patients and other copper-deficiency disorders.

## Experimental Procedures

### Worm Strains, Culture, and RNAi

Bristol N2 was used as the wild-type (WT) *C. elegans* strain. N2 WT, BK014 transgenic worms [*P*_*vha-6*_ *::CUA-1.1::GFP::unc-54 3′ UTR*], BK015 [*Pvha-6::CUA-1.1::GFP::unc-54 3′UTR; cua-1(ok904)*] worms, *chca-1 (tm6506 IV)* and their respective WT broodmates were maintained at 20 °C on NGM plates seeded with *E. coli* OP50 or HT115 (DE3) as a food source. Some worm strains were obtained from the *Caenorhabditis* Genetics Center, which is funded by National Institutes of Health Office of Research Infrastructure Programs (P40 OD010440). RNAi bacteria strains against *chca-1* or *cua-1* were used, and bacteria transformed with the empty L4440 vector were used as a negative RNAi control as described previously (Yuan et al., 2018).

### Elesclomol Treatment in *C. elegans*

Synchronized L1 larval cultures of WT and *chca-1(tm6506)* mutant worms were cultured on basal media or media containing 100 µM BCS plus 10 µM CuCl_2_ or various concentrations of ES for ∼2.5 days. Worms from each condition were analyzed for time of flight (length), extinction (width), and GFP fluorescence using a COPAS Biosort FP-250 (Union Biometrica) to determine body size and copper content by ICP-MS. To measure brood sizes, N2 worms were exposed to *cua-1* (RNAi) or control (*L4440* vector) from synchronized L1 stage with or without 100 µM BCS treatment, supplemented with vehicle (DMSO) or 5 µM ES. After 4 days, worms under each condition were picked to individual plates in triplicate, allowed to lay eggs, and transferred to fresh plates every 24 h for 3 days. Eggs were incubated overnight to allow hatching and progeny number was determined as the total number of hatched larvae. For the survival assay, BK015 transgenic worms were cultured from the L1 stage on RNAi plates supplemented with either vehicle (DMSO) or 5 µM ES for 4 days, and then scored for survival. Animals were considered dead when no signs of viability (movement, pharyngeal pumping, or response to prodding) were detected. The ES concentration required to observe rescue in different tests reflects technical requirements for each assay—e.g., type of bacterial food source, bacterial culture requirements, and the nature of the genetic background or transgene present in the worm strains.

### ICP-MS

Metal content of worms was measured using ICP-MS as described previously (Chun et al., 2017b; Yuan et al., 2018). Values were normalized to the wet weight of worms. Synchronized L1 worms were grown on NGM plates seeded with OP50 supplemented with the indicated amounts of copper, BCS or ES until worms reached the L4 stage. At least three independent biological replicates were analyzed. Copper, and zinc concentrations of mice were measured from nitric acid-digested tissues by ICP-MS as described (Nose et al., 2006). The values were normalized by tissue protein concentration.

### Immunofluorescence

BK014 transgenic worms (*P*_*vha-6*_ *::CUA-1.1::GFP::unc-54 3’ UTR*) were maintained on standard NGM agar plates seeded with OP50 bacteria. Synchronized L1 larvae were cultured on NGM plates with indicated amount of CuCl_2_, vehicle (DMSO) or ES for 3 days. To visualize live worms, animals were paralyzed in M9 buffer containing 10 mM sodium azide (NaN_3_) and mounted on agarose pads. GFP in worms was imaged using an LSM710 confocal microscope (Zeiss).

### Elesclomol Gavage in Mice

The intestine-specific *Ctr1* deletion mouse (*Ctr1* ^*int/int*^) was generated by crossing *Ctr1* ^*flox/flox*^ mice with mice expressing Cre recombinase driven by the *Villin*-promoter as described previously (Nose et al., 2006; Chun et al., 2017b). Age-matched *Ctr1* ^*flox/+*^ or *Ctr1* ^*flox/flox*^ siblings not expressing Cre were served as a control group in this study. All animal procedures were performed in accordance with National Institutes of Health Guide and approved by the Institutional Animal Care and Use Committee at the University of Maryland, College Park (protocol #: R-APR-18-14). Starting from postnatal day (PND) 7, *Ctr1* ^*int/int*^ and control littermates were treated with 10 mg/kg body weight of ES (Selleckchem) or vehicle by gavage every 2 days until PND 31. Mouse body weights were recorded every day from PND 7 to PND 35. Depending on individual mouse weight, an appropriate amount of ES was dissolved in DMSO, and then mixed with 5% methyl cellulose solution (Sigma) to reach a 2% final concentration of ES-DMSO. The vehicle control solution contained 0.5% methyl cellulose solution with 2% DMSO. A subset of mice were dissected at PND 17 for tissue analysis.

### Tissue Preparations and Antibodies

The isolation and extraction of intestinal epithelial cells from mice for immunoblot assays and metal measurements have been described previously (Chun et al., 2017b). The anti-CCS antibody (Santa Cruz Biotechnology) and anti-GAPDH antibody (Sigma) were used at 1:1,000 dilution and 1:5,000 dilution, respectively.

### Statistical Analysis

Statistical analysis was performed with one-way ANOVA followed by Sidak post hoc test, or two-way ANOVA followed by Tukey’s post hoc test, using GraphPad Prism (GraphPad). Differences were considered statistically significant at *p* < 0.05.

## Data Availability

All data presented here are contained within the article.
